# Effect of nutrient deficiencies on *in vitro* Th1 and Th2 cytokine response of peripheral blood mononuclear cells to *Plasmodium falciparum* infection

**DOI:** 10.1186/1475-2875-9-S2-P21

**Published:** 2010-10-20

**Authors:** Erasto V Mbugi, Marjolein Meijerink, Jacobien Veenemans, Prescilla V Jeurink, Matthew McCall, Raimos M Olomi, John F Shao, Jaffu O Chilongola, Hans Verhoef, Huub FJ Savelkoul

**Affiliations:** 1 Cell Biology and Immunology Group, Wageningen University,The Netherlands; 2 Host-Microbe Interactomics,Wageningen University,The Netherlands; 3 Danone Research, Wageningen, The Netherlands; 4 Department of Medical Microbiology, Radboud University, Nijmegen,The Netherlands; 5 Kilimanjaro Christian Medical Centre (KCMC), Moshi, Tanzania; 6 London School of Hygiene and Tropical Medicine, Nutrition and Public Health Intervention Research Unit, London, UK; 7 Muhimbili University of Health and Allied Sciences, Biochemistry Department, School of Medicine, Dar es Salaam, Tanzania

## Background

**Figure 1:**
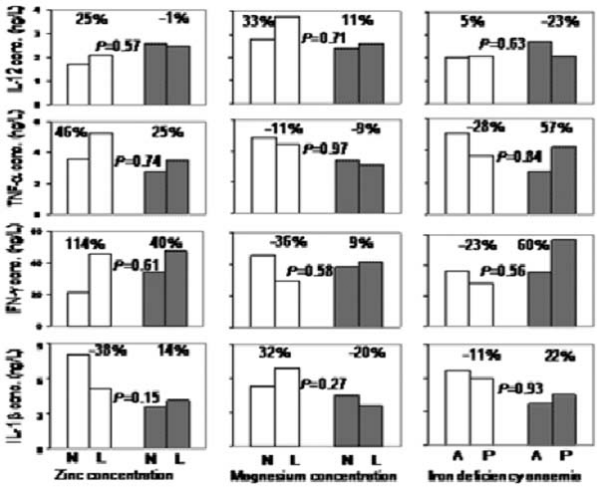
Associations between micronutrient status and supernatant type I cytokine concentrations following 7 days of PEMCs stimulation with *Plasmodium falciparum*-infected erythrocytes by malaria infection status of the child at the time of blood collection. N: nutrient concentrations; L; low concentrations; A: absent; P: present. Percentage indicates paired group difference in cytokine concentrations. Data from children without and with malaria infection at the time of blood collection are indicated with open and shaded bars respectively.

**Figure 2:**
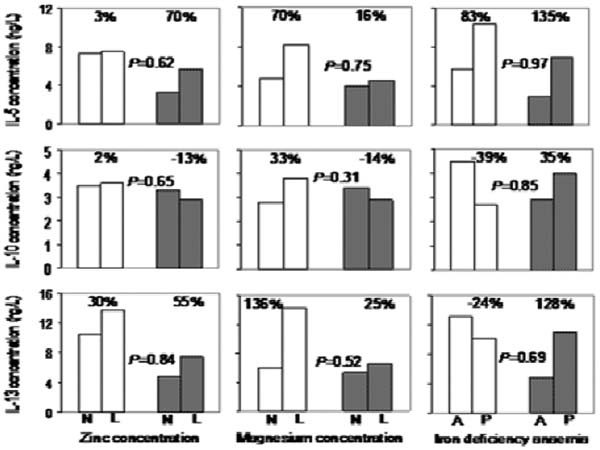
Associations between micronutrient statues and supernatant type II cytokine concentrations following 7 days of PEMCs stimulation with *Plasmodium falciparum*-infected erythrocytes by malaria infection status of the child at the time of blood collection. N: nutrient concentrations; L; low concentrations; A: absent; P: present. Percentage indicates paired group difference in cytokine concentrations. Data from children without and with malaria infection at the time of blood collection are indicated with open and shaded bars respectively.

An appropriate balance between pro-inflammatory and anti-inflammatory cytokines that mediate innate and adaptive immune responses is required for effective protection against human malaria and to avoid immunopathology. In malaria endemic countries, this immunological balance may be influenced by micronutrient deficiencies. Peripheral blood mononuclear cells from Tanzanian preschool children were stimulated *in vitro* with *Plasmodium falciparum*-parasitized red blood cells to determine T-cell responses to malaria under different conditions of nutrient deficiencies and malaria status. The data obtained indicate that zinc deficiency is associated with an increase in TNF response by 37%; 95% CI:14% to 118% and IFN-γ response by 74%; 95% CI: 24% to 297%. Magnesium deficiency, on the other hand, was associated with an increase in production of IL-13 by 80%; 95% CI: 31% to 371% and a reduction in IFN-γ production. These results reflect a shift in cytokine profile to a more type I cytokine profile and cell-cell mediated responses in zinc deficiency and a type II response in magnesium deficiency. The data also reveal a non-specific decrease in cytokine production in children due to iron deficiency anaemia that is largely associated with malaria infection status. Figure 1 and Figure 2.

## Conclusions

The pathological sequels of malaria potentially depend more on the balance between type I and type II cytokine responses than on absolute suppression of these cytokines and this balance may be influenced by a combination of micronutrient deficiencies and malaria status.
